# Postintubation Decline in Oxygen Saturation Index Predicts Mortality in COVID-19: A Retrospective Pilot Study

**DOI:** 10.1155/2021/6682944

**Published:** 2021-05-26

**Authors:** Ala Nozari, Shivali Mukerji, Molly Vora, Alfonso Garcia, Alyssa Park, Nicholas Flores, Robert Canelli, Gerardo Rodriguez, Riccardo Pinciroli, Alexander Nagrebetsky, Rafael Ortega, Sadeq A. Quraishi

**Affiliations:** ^1^Department of Anesthesiology, Boston Medical Center, Boston, MA, USA; ^2^Department of Anesthesia, Critical Care and Pain Medicine, Massachusetts General Hospital, Harvard Medical School, Boston, MA, USA; ^3^Department of Anesthesiology, Tufts Medical Center, Boston, MA, USA

## Abstract

**Background:**

Acute respiratory failure from COVID-19 pneumonia is a major cause of death after SARS-CoV-2 infection. We investigated whether PaO_2_/FiO_2_, oxygenation index (OI), SpO_2_/FiO_2_, and oxygen saturation index (OSI), commonly used to assess the severity of acute respiratory distress syndrome (ARDS), can predict mortality in mechanically ventilated COVID-19 patients.

**Methods:**

In this single-centered retrospective pilot study, we enrolled 68 critically ill mechanically ventilated adult patients with confirmed COVID-19. Physiological variables were recorded on the day of intubation (day 0) and postintubation days 3 and 7. The association between physiological parameters, PaO_2_/FiO_2_, OI, SpO_2_/FiO_2_, and OSI with mortality was assessed using multiple variable logistic regression analysis. Receiver operating characteristic analysis was conducted to evaluate the performance of the predictive models.

**Results:**

The ARDS severity indices were not statistically different on the day of intubation, suggesting similar baseline conditions in nonsurviving and surviving patients. However, these indices were significantly worse in the nonsurviving as compared to surviving patients on postintubation days 3 and 7. On intubation day 3, PaO_2_/FiO_2_ was 101.0 (61.4) in nonsurviving patients vs. 140.2 (109.6) in surviving patients, *p*=0.004, and on day 7 106.3 (94.2) vs. 178.0 (69.3), *p* < 0.001. OI was 135.0 (129.7) in nonsurviving vs. 84.8 (86.1) in surviving patients (*p*=0.003) on day 3 and 150.0 (118.4) vs. 61.5 (46.7) (*p* < 0.001) on day 7. OSI was 12.0 (11.7) vs. 8.0 (10.0) (*p*=0.006) on day 3 and 14.7 (13.2) vs. 6.5 (5.4) (*p* < 0.001) on day 7. Similarly, SpO_2_/FiO_2_ was 130 (90) vs. 210 (90) (*p*=0.003) on day 3 and 130 (90) vs. 230 (50) (*p* < 0.001) on day 7, while OSI was 12.0 (11.7) vs. 8.0 (10.0) (*p*=0.006) on day 3 and 14.7 (13.2) vs. 6.5 (5.4) (*p* < 0.001) on day 7 in the nonsurviving and surviving patients, respectively. All measures were independently associated with hospital mortality, with significantly greater odds ratios observed on day 7. The area under the receiver operating characteristic curve (AUC) for mortality prediction was greatest on intubation day 7 (AUC = 0.775, 0.808, and 0.828 for PaO_2_/FiO_2_, OI, SpO_2_/FiO_2_, and OSI, respectively).

**Conclusions:**

Decline in oxygenation indices after intubation is predictive of mortality in COVID-19 patients. This time window is critical to the outcome of these patients and a possible target for future interventions. Future large-scale studies to confirm the prognostic value of the indices in COVID-19 patients are warranted.

## 1. Introduction

Severe acute respiratory syndrome coronavirus 2 (SARS‐CoV‐2) is known to invade the pulmonary alveolar epithelial cells primarily [1]. While most infected patients have mild to no symptoms, some develop acute hypoxemic respiratory failure that can progress to multiorgan failure and death [2]. Early studies on critically ill patients with coronavirus disease 2019 (COVID-19) revealed long mechanical ventilation times, with one study among New York City patients reporting a median of 18 days and others up to 59 days of mechanical ventilation [3, 4]. Even patients with milder form of the disease may require prolonged respiratory support; one study reported an average of 14.6 days of mechanical ventilation in patients who survived COVID-19 pneumonia [4]. It is important to identify patients who are at greatest risk of major complications so that appropriate treatments are provided earlier in the disease process. This will also aid providers in discussing compassionate palliative care and end of life decisions with the patients and their families. As such, a better understanding of the prognosis and course of the severe coronavirus disease 2019 (COVID-19) is critical for resource allocation and medical management.

An important pathological feature of the lungs obtained on autopsy from patients with COVID-19 is the presence of diffuse alveolar damage and widespread signs of thrombosis, similar to findings in patients with acute respiratory distress syndrome (ARDS) [5, 6]. The clinical presentation and respiratory mechanics of COVID-19 pneumonia are also largely comparable to other forms of ARDS, although a less aggressive “normal compliance” type of COVID-19 has been described [7]. Accordingly, clinicians have applied physiological measures of ARDS severity to COVID-19 patients, without any evidence to support their prognostic values. One important example is the ratio of arterial oxygen tension (PaO_2_) to fraction of inspired oxygen (FiO_2_), or the PaO_2_/FiO_2_ ratio. Besides classifying the severity of ARDS, the PaO_2_/FiO_2_ ratio also has prognostic value, being associated with mortality according to the ARDS Definition Task Force [8]. Mild ARDS (PaO_2_/FiO_2_ 200–300) is associated with 27% mortality, while moderate (PaO_2_/FiO_2_ 100–200) and severe (PaO_2_/FiO_2_ < 100) ARDS are associated with 32% and 45% mortality rates, respectively.

Another physiological index used to stratify the severity of pulmonary dysfunction in ARDS is the product of the mean alveolar pressure (MAP) and the reciprocal of the PaO_2_/FiO_2_ ratio, known as oxygenation index (OI) [9, 10]. OI has also been validated as an independent predictor of mortality in patients with ARDS [11]. Incorporating MAP into its calculation, the OI has the advantage to also reflect alterations in lung mechanics contributing to lung injury. It is routinely used in the care of adult and pediatric patients [12]. However, both PaO_2_/FiO_2_ and OI rely on invasive blood gas analysis. Consequently, oxygenation indices that are based on noninvasive pulse oximetry, namely, the SpO_2_/FiO_2_ ratio and oxygen saturation index (OSI), were also examined and subsequently validated [13–15]. Due to the noninvasive nature of these measurements, they may be of greater clinical value than their invasive counterparts.

Although the clinical and prognostic values of the above oxygenation indices are well established in ARDS, they have yet not been validated in COVID-19 patients. We conducted a retrospective pilot study to explore the association of OI and OSI with survival in a subgroup of COVID-19 patients with significant lung injury and acute respiratory failure requiring mechanical ventilation. Additionally, studying survivors compared to nonsurvivors, we assessed how oxygenation varied over the first 7 days following intubation to establish the value of PaO_2_/FiO_2_, OI, SpO_2_/FiO_2,_ and OSI as early predictors of outcome. We hypothesized that oxygenation indices are better in COVID-19 survivors as compared to nonsurvivors upon intubation and will continue to improve as patients progressively recover.

## 2. Methods

### 2.1. Study Design and Population

The study was conducted at the Boston Medical Center, the largest safety-net hospital in Boston and a level 1 academic trauma center. The study protocol was approved by the Boston Medical Center Institutional Review Board. The requirement for written informed consent was waived given the retrospective nature of the study. All consecutive adult patients with confirmed COVID-19 infection who were admitted to the medical and surgical intensive care units between 3/1/2020 and 6/30/2020 for acute hypoxemic respiratory failure and required mechanical ventilation were enrolled in the study. Patients who were admitted for reasons other than respiratory failure were excluded. Patients who were discharged to another facility, and for whom the primary outcome (survivor vs nonsurvivor) was unknown, were also excluded. We defined day 0 as the day of intubation and initiation of mechanical ventilation.

All patients met the Berlin criteria for ARDS and were treated according to a standard institution-wide, lung-protective ventilation protocol. Specifically, mechanical ventilation was managed by the clinical team of intensivists and respiratory therapists according to the ARDSnet recommendations for titration of PEEP/FiO_2_ and to maintain a plateau pressure of <30 cm H_2_O. Contemporary national guidelines were followed for the management of COVID-19. It included empiric antibiotic treatment with azithromycin, administration of hydroxychloroquine, and immunomodulation with the interleukin-6 receptor inhibitor tocilizumab, interleukin-1 receptor antagonist anakinra, the antiviral agent remdesivir, and anticoagulation, when appropriate. Prone positioning, neuromuscular blocking agents, and inhaled nitric oxide were also employed at the discretion of the treating clinicians.

### 2.2. Data Collection

Demographic data, medical history, preadmission medications, and diagnoses were extracted from each enrolled patient's electronic medical record. Clinical data were also extracted from the electronic medical records until extubation or death, whichever occurred first. Data included patients' vital signs, laboratory parameters, and clinical imaging including chest X-rays. Ventilator settings and arterial blood gas (ABG) parameters were recorded at a daily reference time of approximately 18:00. Special attention was paid to avoiding data collection within 30 minutes of respiratory interventions including suctioning of the endotracheal tube, recruitment maneuvers, bronchoscopies, and acute changes in ventilator settings. Calculated variables included the Acute Physiology and Chronic Health Evaluation II (APACHE II) and Sequential Organ Failure Assessment (SOFA) score, Charlson Comorbidity Index (CCI), respiratory compliance (Crs), PaO_2_/FiO_2_, SpO_2_/FiO_2_, OI, and OSI. OI was calculated as (mean airway pressure × FiO_2_ × 100)/PaO_2_, and OSI was calculated as (mean airway pressure × FiO_2_ × 100)/SpO_2_. Higher OI and OSI values indicate worse oxygenation [11]. Anonymized records were entered into a secure cloud-based data entry online platform (StudyTRAX, Macon, GA) and subsequently extracted for analysis.

### 2.3. Statistical Analysis

The primary outcome variable was death before hospital discharge; therefore, all patients were categorized as survivors or nonsurvivors and followed until death or hospital discharge. Because this was a pilot study, a sample size sufficient to assess the feasibility of the retrospective methods was required as opposed to the global COVID-19 patients. Categorical variables were presented as counts and percentages. Continuous variables were presented as mean ± standard deviation (SD) or median with interquartile range (IQR) and compared between groups (survivors vs nonsurvivors) using *t*-tests or Mann–Whitney U/log-rank tests, respectively. Categorical variables were presented as proportions and compared between groups using chi-squared tests. Independent-samples Kruskal–Wallis test was used to compare the distribution of Crs, PaO_2_/FiO_2_, SpO_2_/FiO_2_, OI, and OSI in surviving and nonsurviving patients, with the Bonferroni correction to preserve the overall type I error at 0.05.

Related-samples Wilcoxon signed rank test was used to compare change in respiratory indices over time. Multivariable logistic regression modeling was used to assess the association between Crs, PaO_2_/FiO_2_, SpO_2_/FiO_2_, OI, and OSI on postintubation days 0, 3, and 7 and in-hospital mortality, adjusting for age, CCI, and sex. Receiver operating characteristic curves were used to analyze prognostic value for mortality for PaO_2_/FiO_2_, SpO_2_/FiO_2_, OI, and OSI on days 0, 3, and 7. Statistical analyses were completed using SPSS Statistics 26 (IBM Corporation, Chicago, IL), SAS (SAS Institute, Cary, NC), and a two-sided significance level of <0.05 was used for statistical inference.

## 3. Results

**Figure 1:**
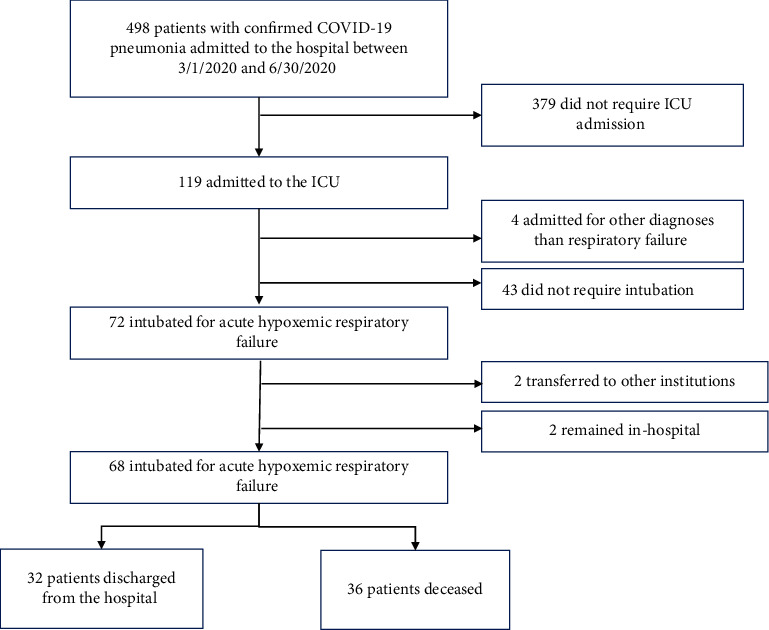
Flow diagram for inclusion of COVID-19 patients in the final analysis.

A total of 498 patients with confirmed COVID-19 pneumonia were admitted to our hospital between March 1, 2020, and June 30, 2020, of whom 119 required ICU admission and 68 met inclusion criteria for this study (Figure 1). These patients had moderate to severe ARDS characterized by a marked impairment in oxygenation with a median PaO_2_/FiO_2_ of 118 [IQR 103.3] in survivors and 105 [IQR 78.1] in nonsurvivors, low compliance (median Crs 18.8 [IQR 18.5] and 16.8 [IQR 7.8], respectively), and bilateral opacities on their chest imaging, which was not different between groups. Thirty-two (47%) patients were successfully extubated and discharged from the hospital, while 36 (53%) expired within 17 [IQR 9] days after hospital admission.

Demographic characteristics and clinical data for each group of patients are summarized in Table 1. Most patients had a variety of comorbid conditions, with the common diagnoses being diabetes mellitus (39% in survivors and 74% in nonsurvivors), coronary artery disease with a history of myocardial infarction (3% in survivors and 28.6% in nonsurvivors), congestive heart failure (12.1% in survivors and 25.7% in nonsurvivors), and cerebrovascular disease and chronic obstructive pulmonary disease. Advanced age, CCI score, history of MI, and preexisting diabetes were associated with an increased risk of mortality, while other clinical characteristics on ICU admission were not. Average ICU length of stay was 13.7 (7.7) in survivors and 15.8 (8.5) in nonsurvivors (*p*=0.365) and hospital length of stay was 21.6 (10.2) and 16.9 (8.7), *p*=0.086, respectively.

Crs, PaO_2_/FiO_2_, SpO_2_/FiO_2_, OI, and OSI were not significantly different between survivors and nonsurvivors on the day of intubation or day 0 (Table 2). PaO_2_/FiO_2_ as well as SpO_2_/FiO_2_ improved in survivors on day 3 (from a PaO_2_/FiO_2_ of 118.1 (103.2) to 140.2 (109.5), *p*=0.029, and a SpO_2_/FiO_2_ of 150 (80) to 210 (90), *p*=0.002), but not in the nonsurviving patients (from 105.0 (78.1) to 101.0 (61.4), *p*=0.245, and from 120 (70) to 130 (90), *p*=0.338, respectively). On postintubation day 7, the PaO_2_/FiO_2_ had increased to 178 (69.3) in survivors, which was markedly better than their PaO_2_/FiO_2_ ratio on the day of intubation (*p*=0.01). Similarly, SpO_2_/FiO_2_ improved to 230 in survivors (*p* < 0.001 versus day 0), but not in nonsurvivors. OI and OSI followed the same trend and continued to worsen (increase) after intubation in the nonsurviving patients. OI increased from 116.0 (90.8) on day 0 to 150.0 (118.4) on day 7, *p*=0.039) while it improved (from 82.9 (95.3) to 61.5 (46.7), *p*=0.013) in survivors. OSI increased from 12.2 (9.2) on day 0 to 14.7 (13.2) on day 7 in nonsurvivors (*p*=0.035) but decreased (improved) in survivors from 10.9 (7.8) to 6.5 (5.4), *p*=0.002.

On postintubation day 3, the Crs and PaO_2_/FiO_2_ and SpO_2_/FiO_2_ were higher and OI and OSI were lower in survivors as compared to nonsurvivors (Table 2). The group difference in these pulmonary variables was even greater on day 7 (*p*=0.007 for Crs and <0.001 for all oxygenation indices), with markedly better (lower) OI and OSI in the survivors as compared to nonsurvivors.

In a multivariate logistic regression model, Crs, PaO_2_/FiO_2_, SpO_2_/FiO_2_, OI, and OSI on intubation days 3 and 7, respectively, were associated with death when adjusted for age, CCI score, and sex (Table 3). Adjusted OR per 10 mmHg decrease in PaO_2_/FiO_2_ were 1.116 (95% CI: 1.020–1.231), *p*=0.0229, and 1.172 (95% CI: 1.051–1.318), *p*=0.0045; adjusted OR per 10 cm H_2_O/mmHg increase in OI were 1.093 (95% CI: 1.010–1.184), *p*=0.0242, and 1.357 (95% CI: 1.138–1.613), *p* < 0.001; adjusted per 10 unit decrease in SpO_2_/FiO_2_ were 1.116 (95% CI: 1.020–1.231), *p*=0.0235, and 1.344 (95% CI: 1.161–1.568), *p* < 0.001; and adjusted OR per cm H_2_O increase in OSI were 1.088 (95% CI: 1.007–1.177), *p*=0.0333, and 1.346 (95% CI: 1.146–1.581), *p* < 0.001, on days 3 and 7 after intubation, respectively.

**Figure 2:**
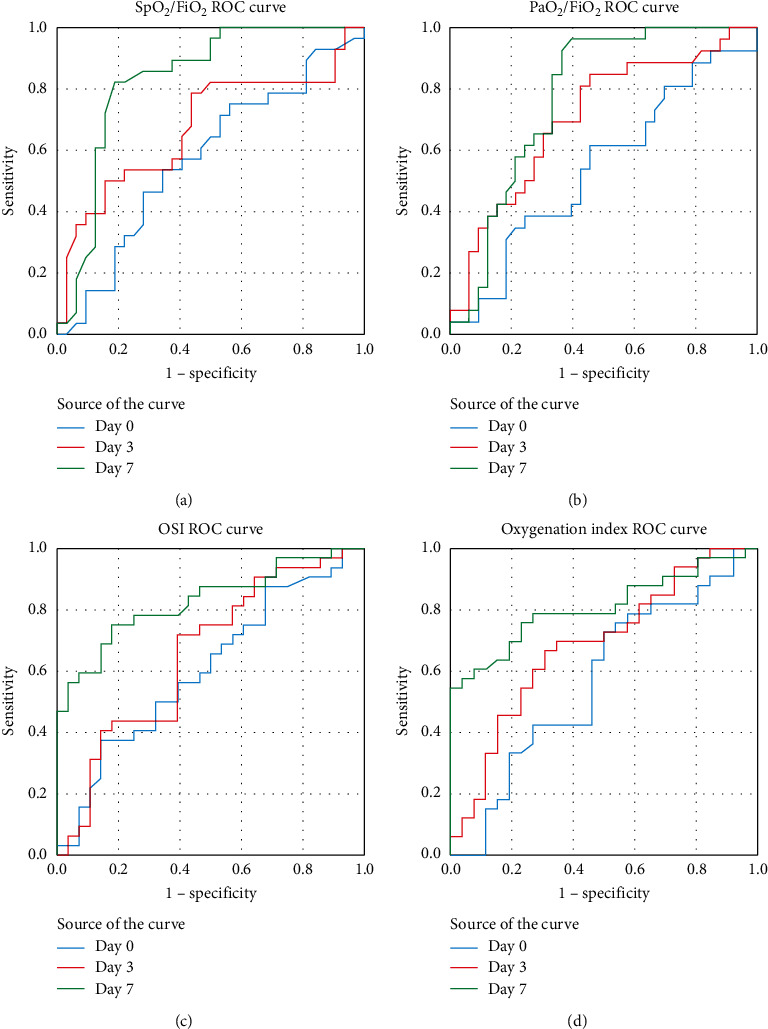
Reviewer operating characteristic (ROC) curves for mortality prediction. Blue: ROC curve for the intubation day 0, red for day 3, and green for day 7.

We tested the individual performance of OI, OSI, PaO_2_/FiO_2_, and SpO_2_/FiO_2_ on intubation days 0, 3, and 7 to predict hospital mortality by calculating the AUC of the receiver operating characteristic curves (Figure 2 and Table 4). While all indices had only moderate performance for mortality prediction on the intubation day (AUC ranging from 0.544 and 0.605), they were significant predictors of mortality on postintubation days 3 and 7 with excellent performance of both the arterial blood gas-dependent variables and the noninvasive pulse oximeter-derived indices: AUC was 0.684 (95% CI: 0.546–0.822) and 0.808 (95% CI: 0.697–0.919) on days 3 and 7 for OI and 0.655 (95% CI: 0.514–0.797) and 0.828 (95% CI: 0.724–0.932) for OSI. Given the report of a better performance of OI and OSI in young patients, we also analyzed the receiver operating characteristic (ROC) curve for patients younger than 60 years of age to determine if discrimination was improved. Although the analysis was limited by the lower number of these patients, the AUC of the ROC curves were even better in this group of younger patients: AUC for OI was 0.802 (95% CI: 0.604–1.000) and 1.000 (95% CI: 1.000–1.000) and for OSI 0.733 (95% CI: 0.517–0.949) and 1.000 (95% CI: 1.000–1.000) on days 3 and 7, respectively.

## 4. Discussion

In this retrospective observational pilot study of 68 critically ill patients with confirmed COVID-19 pneumonia, we present evidence to suggest that PaO_2_/FiO_2,_ SpO_2_/FiO_2_, OI, and OSI can serve as predictors of mortality after intubation. While difference in oxygenation was marginal and did not reach clinical or statistical significance on the day of intubation, suggesting comparable baseline characteristics in survivors and nonsurvivors, survivors rapidly distinguished themselves with a higher PaO_2_/FiO_2_ and SpO_2_/FiO_2_ and lower OI and OSI within 3 days. Lung compliance followed the same trend and was significantly higher in survivors on days 3 and 7 with no significant difference between the two groups on day 0.

### 4.1. The Importance of Prognostication in the Critically Ill COVID-19 Patients

In patients with COVID-19 infection, it is particularly difficult to provide accurate prognostic information, as the case fatality rate has been quite variable ranging from 0.7% in Germany to about 10% in Italy [16]. Decisions about goals of care and life-sustaining treatments are made by incorporating available medical and prognostic data with the preferences of each patient and his or her surrogate. This process depends on the clinician's ability to provide understandable and accurate prognostic information. Existing mortality data must be considered for each specific condition and appropriately applied to the individual patient. Mortality rates between 24% and 67% have been reported in patients requiring hospital admission [17, 18]. COVID-19 patients who require ICU admission and intubation have a particularly high mortality rate, but besides preexisting risk factors such as age, cardiovascular disease, diabetes, and COPD, no clinical prognostic tools have been validated for identifying those at greater risk of death [19, 20]. The findings reported herein confirm the prognostic value of PaO_2_/FiO_2_ and OI in these patients and can therefore serve as an important tool to guide therapeutic management and goals of care discussions and decision-making conversations for COVID-19 patients with respiratory failure. However, clinicians should consider the dynamic nature of the oxygenation indices, and the need to wait at least 3 days after intubation for a more reliable prognostication. These metrics can then serve as additional data points to inform resource allocation in crisis scenarios when access to therapeutic measures is limited or the number of critically ill COVID-19 patients overwhelms the hospitals and health providers.

### 4.2. The Importance of Respiratory Mechanics

Although previous studies have established the importance of oxygenation indices and lung mechanics in patients with ARDS, these important findings have not been validated in COVID-19 patients. The Berlin definition of ARDS uses the PaO_2_/FiO_2_ ratio as the primary measure of ARDS severity, but some studies have failed to validate it as an independent predictor of mortality [21, 22]. It is argued that the outcome in ARDS is affected not only by the degree of oxygenation impairment but also by the mechanical properties of the lungs and the impact of ventilator-induced lung injury (VILI). Integrating both airway pressure and oxygenation into a single index, OI and OSI, might be more powerful predictors of death in ARDS patients [23, 24]. This is of particular importance in COVID-19 pneumonia, given the reported heterogeneity in Crs [7], and respiratory mechanics were therefore expected to have an even greater prognostic relevance. Nevertheless, our results showed that, despite differences in compliance between survivors and nonsurvivors on intubation days 3 and 7, PaO_2_/FiO_2_ and SpO_2_/FiO_2_ remain as powerful as OI and OSI in predicting mortality, possibly suggesting that the primary determinant of mortality is the degree of oxygenation impairment as opposed to respiratory mechanics.

### 4.3. Temporal Evolution of Oxygenation Indices in Intubated COVID-19 Patients

As stated earlier, we were surprised to find that despite differences in baseline clinical characteristics, the ARDS indices (PaO_2_/FiO_2_, OI, and OSI) did not significantly differ between survivors and nonsurvivors on intubation day 0. These findings differ from previous studies of the oxygenation indices, which reported their prognostic power at the time of ARDS diagnosis [15, 25]. It is plausible that, given the initially comparable oxygenation indices in our COVID-19 patients, the pulmonary disease burden may not have been significantly different between the survivor and nonsurvivor groups at the time of intubation. However, over the course of only three days, nonsurvivors were clearly distinguished with significantly worse oxygenation indices as compared to survivors, suggesting significant progression in their pulmonary disease and lung injury. It is of course impossible to determine the cause of the observed divergence in disease progression, but this finding may indicate a time window that is critical to the outcome of intubated COVID-19 patients and potentially a target for future interventions.

### 4.4. The Simplicity of Noninvasive Pulse Oximetry for Assessment of the ARDS Severity

The importance of a continuous assessment of oxygenation is well recognized. Still, it is often limited to recording oxygen saturation or intermittently reviewing blood gas parameters, both of which are critically dependent on the fraction of inspired oxygen. By calculating the PaO_2_/FiO_2_ and SpO_2_/FiO_2_ ratios as well as the oxygenation and oxygen saturation indices, our study extends the role of such physiological indices as relevant estimates of disease severity. Also, it validates these measurements as valuable prognostic tools in critically ill COVID-19 patients. Importantly, we show that noninvasive measures, such as SpO_2_/FiO_2_ and OSI, derived from pulse oximetry, are equally predictive of outcome when compared to those obtained from arterial blood gas values. Moreover, SpO_2_/FiO_2_ is as powerful a predictor as OSI, obviating the need to routinely incorporate pulmonary mechanics in the assessment of the severity of respiratory failure in COVID-19 patients. The effects of therapeutic interventions, as well as prognostication and identification of patients who will benefit the most from continued mechanical ventilation and ICU care, can all be guided by a simple calculation of the SpO_2_/FiO_2_.

### 4.5. Use of SpO_2_/FiO_2_ to Guide Therapeutic Approach to Intubated COVID-19 Patients

While the pathophysiology of ARDS has been extensively studied, its treatment is currently mostly based on supportive intensive care and prevention of VILI. Mortality from the disease ranges from 30 to 60% [11]. The COVID-19-related lung injury is no exception being, at best, only marginally controlled by the currently accepted standard treatments, including antibiotic, antiviral, and immunomodulatory agents. Lung-protective ventilation using small tidal volumes with permissive hypercapnia remains the cornerstone of ARDS management, as it facilitates the goal of minimizing VILI. Lung recruitment strategies, careful optimization of the positive end expiratory pressure (PEEP), pulmonary vasodilators, and prone positioning are also employed to optimize ventilation distribution as well as ventilation-perfusion matching in COVID-19 patients [26]. Evidence to support novel antiviral and anti-inflammatory therapies continues to evolve, as does the data on convalescent plasma, immune globulin, and monoclonal antibodies. The therapeutic and respiratory effects of these treatments might be guided by their impact on pulmonary compliance and oxygenation indices, including a simple calculation of SpO_2_/FiO_2_.

### 4.6. Strengths and Limitations of the Study

The strengths of this study include the unique burden of COVID-19 in the ICU level care at our institution and the uniformity of care provided to these patients. According to the estimates from our admissions office, our institution shouldered a disproportionate number of admissions within our community, with our inpatient census reaching 70% suffering from COVID-19; our ICU census reached 90% of patients with confirmed COVID-19 pneumonia. Though this study has several limitations including a small size and a high mortality rate, the sample size was sufficient to demonstrate the statistically significant and clinically meaningful associations. Unfortunately, given the retrospective nature of this pilot study as well as the limited number of patients, we are unable to demonstrate the clinical significance of our findings. Larger prospective studies are needed to validate our findings and confirm its clinical significance. Our data are also part of a larger dataset collected in several participating academic institutions in Boston and beyond. We aim to validate our findings in this larger cohort.

## 5. Conclusions

Our study validates the prognostic power of the oxygenation indices PaO_2_/FiO_2_, SpO_2_/FiO_2_, OI, and OSI in severely ill COVID-19 patients. Early calculation of these simple metrics can help predict the clinical course of the patient's disease, assist in prognostication, and guide therapeutic interventions.

## Figures and Tables

**Table 1:** Clinical characteristics of the COVID-19 patients with acute hypoxic respiratory failure requiring intubation.

Characteristic	Survivors	Nonsurvivors	*p*
Age in years (SD)	58.1 (16.3)	68.3 (10.8)	0.0036

Gender			
Female (*n* (%))	7 (21.21%)	15 (42.86%)	0.0565
Male (*n* (%))	26 (78.79%)	20 (57.14%)	0.0565

Race			
White (*n* (%))	9 (31.03%)	7 (20.59%)	0.6544
African American (*n* (%))	14 (48.28%)	18 (52.94%)	0.6544
Asian	0 (0%)	1 (2.94%)	0.6544
Others (*n* (%))	0 (0%)	1 (2.94%)	0.6544
Unknown	6 (20.69%)	7 (20.59%)	0.6544

Ethnicity			
Hispanic	14 (42.42%)	9 (25.71%)	0.1455
Non-Hispanic	19 (57.58%)	26 (74.29%)	0.1455
BMI	31.89 (8.43)	33.48 (7.66)	0.4731
SOFA	8 (2.30)	9 (3.42)	0.4768
APACHE II	21.2 (6.8)	20.3 (10.5)	0.8314
Charlson comorbidity index	3.5 (2.9)	5.9 (2.2)	0.0004
Myocardial infarction	1 (3.03%)	10 (28.57%)	0.0043
Congestive heart failure	4 (12.12%)	9(25.71%)	0.1543
Peripheral vascular disease	3 (9.09%)	5 (14.29%)	0.5064
Cerebrovascular disease	5 (15.15%)	4 (11.43%)	0.6507
Chronic obstructive lung disease	3 (9.09%)	6 (17.14%)	0.3275
Liver disease	1 (3.03%)	0 (0%)	0.4821
Chronic kidney disease	2 (6.06%)	10 (28.57%)	0.0149
Diabetes mellitus	13 (39.39%)	26 (74.29%)	0.0036

Laboratory parameters			
C-reactive protein (mg/dL)	119.4 (121.0)	113.8 (110.9)	0.8532
D-dimer (mog/mL)	1.6 (2.4)	3.2 (6.8)	0.2059
Hemoglobin (mg/dL)	12.1 (2.2)	11.9 (1.7)	0.7908
Procalcitonin	1.2 (3.1)	3.0 (5.8)	0.1084
Creatinine (mg/dL)	1.2 (1.3)	1.9 (1.9)	0.0734

Respiratory parameters			
PEEP (cm H_2_O)	10.2 (3.3)	11.8 (3.1)	0.045
FiO_2_ (%)	69.3 (21.7)	73.6 (21.1)	0.4113
MAP (cm. H_2_O)	14.3 (3.5)	16.5 (3.7)	0.0183
Tidal volume (ml)	446.3 (41.7)	420.5 (65.7)	0.0565
Days ventilated	13.9 (9.1)	14.7 (8.3)	0.7304
ICU length of stay (days)	13.7 (7.7)	15.8 (8.5)	0.3652
Hospital length of stay (days)	21.6 (10.2)	16.9 (8.7)	0.0859

Values are presented as mean (%), mean (SD) when normally distributed, or median (IQR).

**Table 2:** ARDS severity indices for COVID-19 survivors and nonsurvivors on days 0, 3, and 7 after intubation.

	Intubation day	Survivors	Nonsurvivors	*p*
Lung compliance	Day 0	18.8 (IQR 18.5)	16.8 (IQR 7.8)	0.105
	Day 3	19.4 (IQR 22.4)	14.6 (IQR 7.2)	0.014
	Day 7	21.5 (IQR 25.3)	15.8 (IQR 9.9)	0.007

PaO_2_/FiO_2_ ratio	Day 0	118.1 (IQR 103.3)	105.0 (IQR 78.1)	0.432
	Day 3	140.2 (IQR 109.6)	101.0 (IQR 61.4)	0.004
	Day 7	178.0 (IQR 69.3)	106.3 (IQR 94.2)	<0.001

Oxygenation index	Day 0	82.9 (IQR 95.3)	116.0 (IQR 90.8)	0.165
	Day 3	84.8 (IQR 86.1)	135.0 (IQR 129.7)	0.003
	Day 7	61.5 (IQR 46.7)	150.0 (IQR 118.4)	<0.001

SpO_2_/FiO_2_ ratio	Day 0	150 (IQR 80)	120 (IQR 70)	0.217
	Day 3	210 (IQR 90)	130 (IQR 90)	0.003
	Day 7	230 (IQR 50)	130 (IQR 90)	<0.001

Oxygen saturation index	Day 0	10.9 (IQR 7.8)	12.2 (IQR 9.2)	0.052
	Day 3	8.0 (IQR 10.0)	12.0 (IQR 11.7)	0.006
	Day 7	6.5 (IQR 5.4)	14.7 (IQR 13.2)	<0.001

Data are presented as median (interquartile range).

**Table 3:** Unadjusted and multivariate adjusted odds ratio of death for Crs, PaO_2_/FiO_2_, OI, SpO_2_/FiO_2_, and OSI.

Day 0	Unadjusted OR (95% CI)	Multivariate OR ^*∗*^ (95% CI)	*p* val (unadj)	*p* val (adj)
Crs (per 10 ml/cm H_2_O decrease)	1.644 (0.951, 2.839)	1.318 (0.960, 2.456)	0.0737	0.383
PaO_2_/FiO_2_ (per 10 mmHg decrease)	1.020 (0.951, 1.083)	1.010 (0942, 1.083)	0.6193	0.8289
OI (per 10 cm H_2_O/mmHg increase)	1.041 (0.961, 1.116)	1.030 (0.942, 1.12)	0.3593	0.5039
SpO_2_/FiO_2_ (per 10 unit decrease)	1.051 (0.961, 1.161)	1.062 (0.961, 1.172)	0.3908	0.2285
OSI (per cm H_2_O increase)	1.107 (0.996, 1.230)	1.119 (0.996, 1.258)	0.0587	0.0588

Day 3	Unadjusted OR (95% CI)	Multivariate OR ^*∗*^ (95% CI)	*p* val (unadj)	*p* val (adj)

Crs (per 10 ml/cm H_2_O decrease)	1.930 (1.149, 3.219)	1.708 (1.00, 2.943)	0.0124	0.0512
PaO_2_/FiO_2_ (per 10 mmHg decrease)	1.104 (1.020, 1.207)	1.116 (1.020, 1.231)	0.0164	0.0229
OI (per 10 cm H_2_O/mmHg increase)	1.093 (1.020, 1.207)	1.0937 (1.010, 1.184)	0.0142	0.0242
SpO_2_/FiO_2_ (per 10 mmHg decrease)	1.138 (1.041, 1.243)	1.116 (1.020, 1.231)	0.0048	0.0235
OSI (per cm H_2_O increase)	1.092 (1.017, 1.173)	1.088 (1.007, 1.177)	0.0156	0.0333

Day 7	Unadjusted OR (95% CI)	Multivariate OR ^*∗*^ (95% CI)	*p* val (unadj)	*p* val (adj)

Crs (per 10 ml/cm H_2_O decrease)	2.119 (1.207, 3.772)	2.004 (1.072, 3.740)	0.0094	0.0282
PaO_2_/FiO_2_ (per 10 mmHg decrease)	1.149 (1.041, 1.268)	1.172 (1.051, 1.318)	0.0053	0.0045
OI (per 10 cm H_2_O/mmHg increase)	1.267 (1.105, 1.452)	1.357 (1.138, 1.613)	0.0006	0.0006
SpO_2_/FiO_2_ (per 10 unit decrease)	1.293 (1.138, 1.466)	1.344 (1.161, 1.568)	<0.0001	0.0001
OSI (per cm H_2_O increase)	1.275 (1.120, 1.451)	1.346 (1.346, 1.581)	0.0002	0.0003

**Table 4:** Comparison of areas under the receiver operating characteristic curve for discrimination of hospital survival.

Day 0	AUC	95% CI
Lung compliance	0.584	(0.421, 0.748)
PaO_2_/FiO_2_	0.544	(0.394, 0.694)
Oxygenation index	0.569	(0.417, 0.721)
SpO_2_/FiO_2_	0.573	(0.426, 0.719)
Oxygen saturation index	0.605	(0.461, 0.750)

Day 3	AUC	95% CI

Lung compliance	0.625	(0.459, 0.790)
PaO_2_/FiO_2_	0.708	(0.573, 0.843)
Oxygenation index	0.684	(0.546, 0.822)
SpO_2_/FiO_2_	0.674	(0.532, 0.816)
Oxygen saturation index	0.655	(0.514, 0.797)

Day 7	AUC	95% CI

Lung compliance	0.680	(0.525, 0.835)
PaO_2_/FiO_2_	0.775	(0654, 0.896)
Oxygenation index	0.808	(0.697, 0.919)
SpO_2_/FiO_2_	0.830	(0.721, 0.939)
Oxygen saturation index	0.828	(0.724, 0.932)

## Data Availability

The patient data used to support the findings of this study are restricted by the BMC and MGH Institutional Review Boards in order to protect patient privacy. Data are available from Drs. Nozari or Pinciroli for researchers who meet the criteria for access to confidential data.
